# Large Language Models for Epidemiological Research via Automated Machine Learning: Case Study Using Data From the British National Child Development Study

**DOI:** 10.2196/43638

**Published:** 2023-09-19

**Authors:** Rasmus Wibaek, Gregers Stig Andersen, Christina C Dahm, Daniel R Witte, Adam Hulman

**Affiliations:** 1 Steno Diabetes Center Copenhagen, Herlev, Denmark; 2 Department of Public Health, Aarhus University, Aarhus, Denmark; 3 Steno Diabetes Center Aarhus, Aarhus University Hospital, Aarhus, Denmark

**Keywords:** natural language processing, deep learning, language model, epidemiology, cohort study, prediction, NLP, prediction model, child development, regression model, large language model, LLM

## Abstract

**Background:**

Large language models have had a huge impact on natural language processing (NLP) in recent years. However, their application in epidemiological research is still limited to the analysis of electronic health records and social media data.

**Objectives:**

To demonstrate the potential of NLP beyond these domains, we aimed to develop prediction models based on texts collected from an epidemiological cohort and compare their performance to classical regression methods.

**Methods:**

We used data from the British National Child Development Study, where 10,567 children aged 11 years wrote essays about how they imagined themselves as 25-year-olds. Overall, 15% of the data set was set aside as a test set for performance evaluation. Pretrained language models were fine-tuned using AutoTrain (Hugging Face) to predict current reading comprehension score (range: 0-35) and future BMI and physical activity (active vs inactive) at the age of 33 years. We then compared their predictive performance (accuracy or discrimination) with linear and logistic regression models, including demographic and lifestyle factors of the parents and children from birth to the age of 11 years as predictors.

**Results:**

NLP clearly outperformed linear regression when predicting reading comprehension scores (root mean square error: 3.89, 95% CI 3.74-4.05 for NLP vs 4.14, 95% CI 3.98-4.30 and 5.41, 95% CI 5.23-5.58 for regression models with and without general ability score as a predictor, respectively). Predictive performance for physical activity was similarly poor for the 2 methods (area under the receiver operating characteristic curve: 0.55, 95% CI 0.52-0.60 for both) but was slightly better than random assignment, whereas linear regression clearly outperformed the NLP approach when predicting BMI (root mean square error: 4.38, 95% CI 4.02-4.74 for NLP vs 3.85, 95% CI 3.54-4.16 for regression). The NLP approach did not perform better than simply assigning the mean BMI from the training set as a predictor.

**Conclusions:**

Our study demonstrated the potential of using large language models on text collected from epidemiological studies. The performance of the approach appeared to depend on how directly the topic of the text was related to the outcome. Open-ended questions specifically designed to capture certain health concepts and lived experiences in combination with NLP methods should receive more attention in future epidemiological studies.

## Introduction

Understanding human language is not a trivial task for machines. Natural language processing (NLP), that is, the analysis of free text with computational methods, has existed as a scientific field for more than half a century [1]. The introduction of large language models was a major leap for the field around the millennium [2]. The essentials of NLP have been reviewed recently with a clinical target audience in mind [3]. Text can be considered as a sequence of characters or words. These linguistic building blocks are referred to as tokens. Once a text is parsed into tokens, a mathematical representation is generated. The most common approach is to use word embeddings—mapping tokens to numerical vectors. These embeddings are trained (assessed in a data-driven manner) on large data sets, and their key feature is that they preserve the relationships between related words. Transformers, introduced in 2017 [4], are currently the most popular underlying model architecture as they excel at contextualizing words in sentences. The vast amount of easily accessible textual data on the internet represents a massive resource for training language models. As compared to supervised machine learning (ML) approaches where the outcome (target) is available in the training data set, the situation is more complicated with language modeling, where the assignment of labels is not always straightforward. One popular approach, also applied in one of the most influential language models—Bidirectional Encoder Representations from Transformers (BERT) [5], is masked language modeling, that is, masking a certain proportion of words and considering them as outcomes to be predicted based on the preceding sequences of words. Another approach used in the development of BERT is the prediction of the next sentence in a text out of several options. This is a semisupervised training strategy that makes it possible to turn vast amounts of texts, for example, the English-language Wikipedia corpus, into a training set for a language model [5]. Technological advancements in computational tools (eg, graphical processing units and parallelization) have allowed language models to increase massively in size to hundreds of billions of parameters in recent years and have pushed performance closer and closer to human level in various NLP tasks [5-7]. These language models, developed by tech giants or their subsidiaries, are used in search engines, language translators, and auto-correct functions, among others, affecting our everyday lives.

Large language models have a broad scientific potential as well, and with the advent of transfer learning, they are more and more available for those who do not necessarily have the computational resources of tech giants. Transfer learning is the reuse of a pretrained model for a new data set or even a new prediction task that is different from the one it was originally trained for [8]. This approach unlocks the potential of ML for smaller studies by using knowledge representations (in a form of pretrained parameters) learned in large data sets. The significance of the method for NLP was first demonstrated by Howard and Ruder [9], who improved the predictive performance on several NLP benchmarks by ~30% by training a universal language model and reused it for specialized tasks via transfer learning. Even though transfer learning broadens the group of potential users of large language models and deep learning in general, it still requires specialized skills to apply these models. Web services to automate the training and deployment of ML models (automated ML [AutoML]) have been developed to overcome this barrier and unlock the potential in deep learning for researchers without specialized ML skills; however, their use is not common in the clinical research community [10,11].

In addition to knowledge identification (named-entity recognition), synthetization, or discovery in the scientific literature [12], NLP has had an impact on clinical research with applications mostly focusing on the analysis of electronic health records or social media data [13,14], most likely due to the large size of these data sources. However, the potential in free-text data and NLP are to date not fully exploited in classical epidemiological studies. It is likely that NLP performs better than classical regression prediction models in certain settings, but not all, depending on the content of the input text and the outcome to be predicted.

We designed a case study to evaluate the performance of large language models, trained via AutoML, in predicting current reading comprehension and future BMI and physical activity based on essays written by 11-year-old children about how they imagine themselves as 25-year-olds. We then compared this with a classical regression approach, including demographic and lifestyle factors that were selected based on prior domain knowledge as predictors. We explicitly aimed to study and compare the predictive ability of the models (accuracy or discrimination), without the consideration of etiology as it is only on this benchmark that ML and traditional models can currently be compared.

## Methods

### Data Source

The National Child Development Study (NCDS) originally included 17,415 individuals born in the same week of 1958 in England, Wales, or Scotland [15]. In a total of 12 sweeps, cohort members have been followed since then via interviews, surveys, and biomedical measurements, mostly focusing on health and sociodemographic information not only of the participants but also to some extent their parents. In this study, we used information from baseline (at birth in 1958), sweep 1 (age 7 years in 1965), sweep 2 (age 11 years in 1969), and sweep 5 (age 33 years in 1991) [16-18].

The three outcomes are (1) reading comprehension score (continuous) at age 11 years; (2) BMI (continuous) at age 33 years; and (3) physical activity (binary) at age 33 years. Reading comprehension (score range: 0-35) was assessed using a test filled out at school. The original test is available on the web on the UK Data Service portal [17]. BMI was calculated as weight (kg) divided by height (m) squared based on anthropometric measurements taken at the time of the interview. Physical activity was assessed with 2 questions, asking whether participants do any sport or exercise, and if so, how often. Participants were considered as physically active if they reported exercising at least once a week.

At the age of 11 years, the children were asked to write an essay about how they imagined themselves as 25-year-olds [16]. The instructions were the following: “Imagine that you are 25 years old now. Write about the life you are leading, your interests, your home life and your work at the age of 25. (You have 30 min to do this.).” Out of the 13,669 essays, 10,567 (77.31%) were transcribed [19], which served as the input for the deep learning analyses.

We had access to the following variables that were available at the birth of the participants: sex, ethnicity, birth weight, gestational age at birth, parity, age and BMI of the mother and father, whether the mother spoke English at home, mother’s smoking habit prior to pregnancy, and social class of the head of the household. Moreover, there was information available on the children’s eating habits at age 7 years (appetite and overeating) and BMI, lifestyle (how often they read books, used parks, and did sports activities), and general ability score (similar to an IQ test) at age 11 years. These variables, selected based on prior knowledge in relation to the outcomes, are only a minor subset of those available in the cohort. Extensive descriptions of the different sweeps of the study are available on the web on the UK Data Service portal [16-18].

### Predictive Modeling and Performance Evaluation Strategy

An analytical sample was defined for each of the 3 outcomes. A random sample of approximately 15% of the participants was reserved as a test set in each of the 3 analytical samples before developing the models, and the remaining 85% constituted the development set. In the AutoML approach, the development set was further split into a training set (80%) and a validation set (20%). All reported performance metrics were evaluated on the test sets.

The root mean square error (RMSE) was used as a performance metric for the continuous outcomes, that is, reading comprehension and BMI. Additionally, 95% CIs for RMSE were calculated using the basic bootstrap method with the *boot* package (version 1.3-28) in R (The R Foundation for Statistical Computing). To provide a benchmark RMSE score for comparison, we applied and evaluated a naive approach, that is, assigned the mean value of the outcome from the development data set as predictions in the test set.

Discrimination, measured by the area under the receiver operating characteristics curve (AUC ROC), was used as a performance metric for the binary outcome: physical activity. The naive benchmark was random assignment, and thus, an AUC ROC of 0.5 was defined.

### Classical Approach: Regression Models

Regression models included predefined sets of variables that could vary for the 3 outcomes based on prior epidemiological knowledge. Models were fitted using the entire development set after applying multiple imputation (within the development set for each particular outcome) by chained equations to impute missing predictors (*mice* R package; version 3.14.0). We generated 10 imputed samples with the maximum number of iterations set to 30. Estimates were then pooled from the 10 resulting models. The *mice* models derived in the development sets were subsequently applied to the test sets to avoid information leakage.

Reading comprehension score and BMI were modeled using linear regression. For the reading comprehension outcome, we fitted 2 models, with and without including the general ability score among the predictors. The binary outcome physical activity at age 33 years was modeled with logistic regression. The complete list of variables included in each model are shown in Tables 1 and 2.

**Table 1.** Linear regression coefficients from prediction models for reading comprehension score and BMI.

Predictor	Reading comprehension score at age 11 years (n=8890)			BMI at age 33 years (n=6010)	
	Imputed, n (%)	Model 1 coefficients (95% CI)	Model 2 coefficients (95% CI)	Imputed, n (%)	Model coefficients (95% CI)
Sex (reference: male)	0	–0.07 (–0.31 to 0.18)	–0.63 (–0.81 to –0.45)	0	–1.1 (–1.3 to –0.9)
**Ethnicity (reference: European)**	1358 (15.28)			794 (13.21)	
African		–3.20 (–4.41 to –2.00)	–0.60 (–1.41 to 0.22)		1.62 (0.27 to 2.97)
Asian		–2.90 (–4.43 to –1.37)	–1.40 (–2.41 to –0.38)		1.11 (–0.26 to 2.49)
Mother’s age (10 years)	423 (4.76)	1.59 (1.23 to 1.95)	0.92 (0.66 to 1.18)	234 (3.89)	–0.22 (–0.56 to 0.12)
Father’s age (10 years)	732 (8.23)	0.53 (0.22 to 0.84)	0.37 (0.16 to 0.59)	415 (6.91)	–0.19 (–0.48 to 0.10)
Mother’s BMI	N/A^a^	N/A	N/A	615 (10.23)	0.12 (0.09 to 0.14)
Father’s BMI	N/A	N/A	N/A	752 (12.51)	0.10 (0.06 to 0.14)
Mother does not speak English at home	1015 (11.42)	–0.06 (–0.46 to 0.34)	–0.14 (–0.42 to 0.14)	N/A	N/A
Birth weight (100 g)	694 (7.81)	0.11 (0.08 to 0.13)	0.03 (0.01 to 0.05)	414 (6.89)	0.00 (–0.03 to 0.02)
Gestational age	1267 (14.25)	0.00 (–0.02 to 0.01)	0.00 (–0.01 to 0.01)	764 (12.71)	0.00 (–0.01 to 0.01)
**Parity (reference: nulliparous)**	421 (4.74)			232 (3.86)	
Primiparous		–1.33 (–1.64 to –1.03)	–0.72 (–0.94 to –0.49)		–0.12 (–0.38 to 0.14)
Multiparous		–3.45 (–2.81 to –1.48)	–1.53 (–1.78 to –1.27)		0.19 (–0.11 to 0.50)
Maternal smoking	450 (5.06)	–0.41 (–0.66 to –0.17)	0.02 (–0.15 to 0.20)	250 (4.16)	0.38 (0.17 to 0.58)
**Socioeconomic status (reference: I)**	945 (10.63)			562 (9.35)	
II		–1.24 (–1.85 to –0.63)	–0.27 (–0.72 to 0.18)		–0.30 (–0.80 to 0.20)
III (nonmanual)		–2.14 (–2.81 to –1.48)	–0.56 (–1.05 to –0.08)		0.03 (–0.56 to 0.62)
III (manual)		–4.17 (–4.73 to –3.60)	–1.34 (–1.7 to –0.92)		0.32 (–0.16 to 0.79)
IV		–5.11 (–5.72 to –4.49)	–1.68 (–2.15 to –1.22)		0.26 (–0.27 to 0.79)
V		–6.39 (–7.14 to –5.65)	–1.89 (–2.45 to –1.33)		0.54 (–0.12 to 1.20)
No male head of household		–4.64 (–5.45 to –3.83)	–1.46 (–2.04 to 0.88)		0.27 (–0.39 to 0.93)
Poor appetite	N/A	N/A	N/A	607 (10.1)	–0.15 (–0.43 to 0.13)
Overeating	N/A	N/A	N/A	612 (10.18)	0.06 (–0.39 to 0.52)
**Reading books (reference: often)**	276 (3.1)			N/A	
Sometimes		–0.61 (–0.86 to –0.36)	–0.20 (–0.39 to –0.02)		N/A
Hardly ever		–2.27 (–2.71 to –1.83)	–0.72 (–1.04 to –0.41)		N/A
**Sport (reference: often)**	238 (2.68)			146 (2.43)	
Sometimes		0.09 (–0.17 to 0.34)	–0.07 (–0.25 to 0.12)		–0.15 (–0.38 to 0.08)
Hardly ever		–0.06 (–0.47 to 0.35)	0.10 (–0.19 to 0.39)		–0.07 (–0.41 to 0.27)
**Park use (reference: often)**	847 (9.53)			488 (8.12)	
Sometimes		0.38 (0.12 to 0.65)	0.10 (–0.10 to 0.30)		–0.30 (–0.54 to –0.06)
Never		0.36 (–0.15 to 0.87)	0.32 (–0.04 to 0.69)		0.05 (–0.44 to 0.54)
Not available		0.06 (–0.34 to 0.46)	–0.04 (–0.33 to 0.25)		–0.11 (–0.45 to 0.24)
General ability score	1 (0.01)	N/A	0.26 (0.26 to 0.27)	N/A	N/A
BMI at age 11 years	N/A	N/A	N/A	886 (14.74)	0.67 (0.62 to 0.72)

a N/A: not applicable.

**Table 2.** Odds ratios (OR) from the prediction model for physical activity.

Predictor		Outcome: physical activity at age 33 years (n=6204)	
		Imputed, n (%)	OR (95% CI)
Sex (reference: male)		0 (0)	1.13 (1.01-1.27)
**Ethnicity (reference: European)**		846 (14.04)	
	African		0.68 (0.34-1.35)
	Asian		0.76 (0.40-1.45)
Mother’s age (10 years)		234 (3.88)	0.98 (0.83-1.15)
Father’s age (10 years)		431 (7.15)	1.05 (0.91-1.21)
Mother’s BMI		652 (10.82)	0.98 (0.97-1.00)
Father’s BMI		809 (13.43)	0.99 (0.98-1.01)
Birth weight (100 g)		411 (6.82)	1.00 (0.99-1.01)
Gestational age		796 (13.21)	1.00 (0.99-1.01)
**Parity (reference: nulliparous)**		232 (3.85)	
	Primiparous		0.99 (0.86-1.13)
	Multiparous		0.94 (0.80-1.11)
Maternal smoking		253 (4.2)	0.92 (0.81-1.04)
**Socioeconomic status (reference: I)**		579 (9.61)	
	II		0.91 (0.69-1.19)
	III (nonmanual)		0.83 (0.61-1.12)
	III (manual)		0.82 (0.63-1.06)
	IV		0.77 (0.59-1.02)
	V		0.64 (0.45-0.92)
	No male head of household		0.72 (0.51-1.02)
Poor appetite		631 (10.47)	0.87 (0.71-1.05)
Overeating		630 (10.46)	0.92 (0.74-1.15)
**Sport (reference: often)**		148 (2.46)	
	Sometimes		0.90 (0.79-1.02)
	Hardly ever		0.70 (0.59-0.84)
**Park use (reference: often)**		510 (8.47)	
	Sometimes		0.97 (0.85-1.10)
	Never		0.77 (0.61-0.97)
	Not available		0.73 (0.59-0.90)
BMI at age 11 years		934 (15.50)	1.01 (0.97-1.04)

### Deep Learning Approach: NLP Using Large Language Models

We used an AutoML tool, AutoTrain by Hugging Face [20], to develop our NLP prediction model. AutoTrain is a web-based service to train and deploy state-of-the-art ML models (text or tabular as of June 2022). The data sets were uploaded as comma-separated values files including 2 columns: the essays (as text) and the outcome. AutoTrain then split this data set into a training set (80%) and a validation set (20%) and started training (fine-tuning) a variety of pretrained large language models. The number of models can be defined by the user. We chose n=15 for this study. After the training process for all 15 models was complete, we accessed the best-performing model through Hugging Face’s application programming interface from Python (Python Software Foundation) and evaluated predictive performance on the reserved 15% in the test set. We did this for all 3 outcomes.

### Ethical Considerations

We analyzed a publicly available, anonymized data set; therefore, our study did not require ethical approval.

## Results

### Reading Comprehension Score (Age 11 Years)

Out of 10,567 participants with transcribed essays, 10,490 (99.27%) completed the reading comprehension test, forming the analytical sample for this outcome. From the 10,490 participants, a random sample of 1600 (15.25%) participants were set aside for testing (test set), leaving data from 8890 (84.75%) participants for model development (development set). Reading comprehension scores ranged from 0 to 34, with a median value of 16 (IQR 12-20). The distribution was similar in the test set and only differed slightly in the maximum (35) and the upper-quartile (21) values.

**Figure 1.**
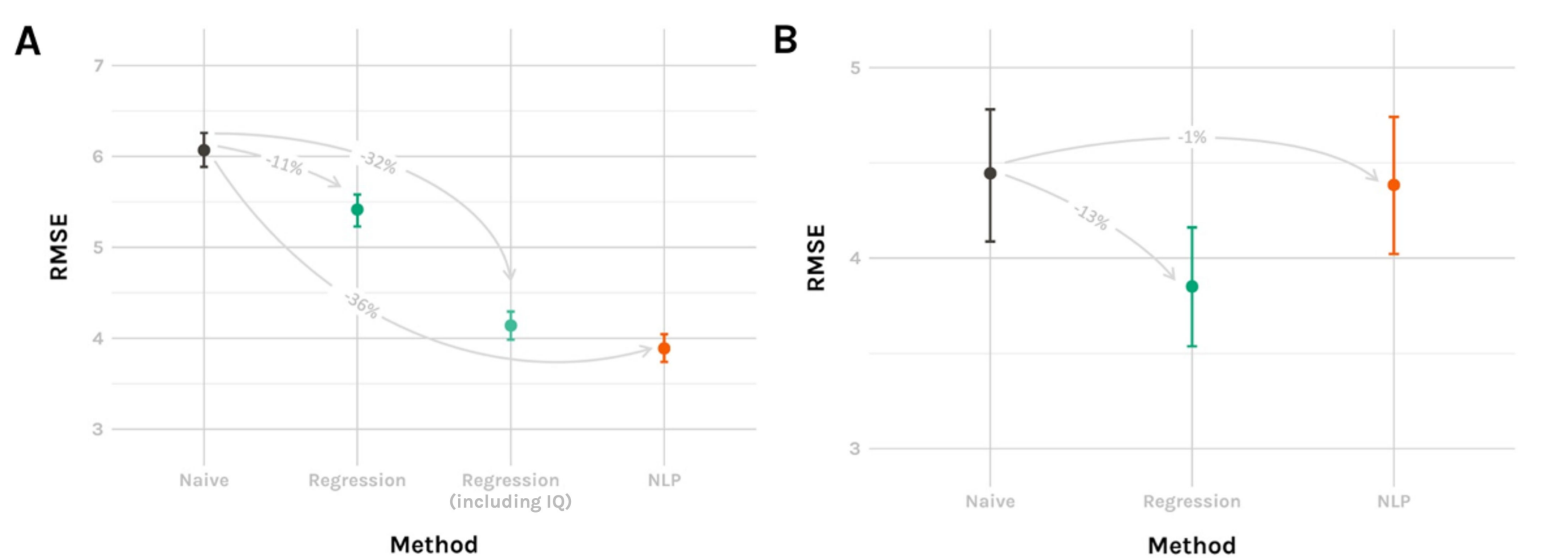
Performance of the prediction models versus the benchmark approach (naive prediction: assignment of the mean value from the training set) for (A) reading comprehension score and (B) BMI. Root mean square errors (RMSEs) are presented with 95% CIs. Percentages represent differences compared to the benchmark approach. NLP: natural language processing.

The main results are shown in Figure 1. The naive benchmark had an RMSE of 6.07 (95% CI 5.89-6.26), which was outperformed by both the classical regression and the deep learning approach. The linear regression model without the general ability score had an 11% better performance than the naive benchmark with an RMSE of 5.41 (95% CI 5.23-5.58). This was further improved when including the general ability score in the model (4.14, 95% CI 3.98-4.30). The best performance and thus lowest RMSE was achieved by the deep learning approach (3.89, 95% CI 3.74-4.05), corresponding to a 36% lower RMSE than the naive benchmark.

The linear regression models revealed that several predictors were associated with the reading comprehension score. Male sex, European ethnicity, having older parents, being the first child in the family, higher birth weight, higher socioeconomic status, reading books often, and having a higher general ability score were all positively associated with reading comprehension. Regression coefficients are presented in Table 1.

### BMI (Age 33 Years)

The analytical sample for the BMI analysis consisted of 7060 participants who later had their weight and height measured at age 33 years. From the 7060 participants, a random sample of 1050 (14.87%) participants were set aside for testing model performance, leaving 6010 (85.13%) participants in the development set. BMI values ranged from 12.3 to 50.6 kg/m^2^ in the development set and from 15.0 to 50.8 kg/m^2^ in the test set. Median values were similar: 24.3 (IQR 22.3-27.1) and 24.4 (IQR 22.2-26.8) kg/m^2^, respectively.

Performance metrics are shown in Figure 1. The naive benchmark had an RMSE of 4.45 (95% CI 4.09-4.78), which was similar to the performance of the deep learning approach (4.38, 95% CI 4.02-4.74). The regression model performed ~13% better, achieving an RMSE of 3.85 (95% CI 3.54-4.16).

Several variables were associated with BMI at age 33 years according to the regression model, including sex, ethnicity, parental BMI, parity, maternal smoking before pregnancy, use and access to parks, and BMI at age 11 years. Regression coefficients are presented in Table 1.

### Physical Activity (Age 33 Years)

We had information on physical activity at age 33 years from 7304 participants. We selected 1100 (15.06%) of them randomly for the test set, leaving 6204 (84.94%) participants for model development. Overall, 68.75% (4265/6204) and 69.55% (765/1100) were physically active in the development and test sets, respectively. The logistic regression and NLP approaches had the same performance (AUC ROC=0.55, 95% CI 0.52-0.60), representing poor discriminatory power. There were a few variables associated with the outcome in the logistic regression model: sex, socioeconomic status, mother’s BMI, sport activities, and use or access to parks at age 11 years. Odds ratios are presented in Table 2.

## Discussion

Our study demonstrated the potential of using deep learning–based large language models for text prediction in epidemiological studies and compared it to classical statistical methods. We observed different rankings of predictive performance between the deep learning and classical approaches across the 3 outcomes. The performance of the deep learning approach appeared to depend on how closely the actual task, that is, writing an essay about the future, was related to the outcome. Writing and reading skills among children are expected to be associated with each other, so the language model could have picked up on linguistic features such as grammatical correctness, vocabulary, complexity of sentences, etc, which led to the NLP method clearly outperforming linear regression when predicting the reading comprehension score. This was still true when the general ability score was added to the regression model as a predictor, despite its high correlation with reading comprehension. However, this performance came with a computational price. Large language models include hundreds of millions or even billions of parameters, whereas our regression model included 26. In addition to simplicity, interpretability is another positive feature of linear regression. The model revealed several strong predictors and quantified associations via interpretable regression coefficients, for example, a social gradient with about a 5-point estimated difference between the highest and lowest socioeconomic classes. Although the coefficients are expressed in easily understandable units, they should not be interpreted in the etiological sense, unless a causal framework is applied. With the increasing interest in ML and causal inference, the development of ML methods integrating causal structures is warranted [21].

Epidemiologists and clinicians are comfortable with interpreting the usual measures of association: linear regression coefficients, odds ratios, or hazard ratios. Although we are far from understanding the overall nature of large language models, there are emerging methods in explainable artificial intelligence (AI) that can help to understand the driving factors of at least individual predictions (eg, which features or specific expression in a text led to a prediction). However, they are yet to be integrated into AutoML tools. Access to explainable AI tools (eg, LIME [22]) as part of AutoML solutions is likely to contribute to a more widespread use of deep learning in epidemiological research, where we often ask etiological questions and predictive performance is not necessarily the main focus.

Children were directly asked about their interests as 25-year-olds as part of the essay task, which could potentially include information on physical activity. We therefore expected a similar performance for the NLP and regression approaches. Both approaches picked up some signals in the data, demonstrated by discrimination nominally exceeding random assignment (AUC ROC=0.5), but their performance was still poor and statistically not different from each other. A previous study from the NCDS reported that 42% of boys and 34% of girls mentioned physical activity in their essays [19]. The authors then used this information to predict their physical activity patterns during adulthood, and they found a positive association among boys, but not girls. Pongiglione et al [19] used a 2-step approach: first, they applied a supervised ML method (support vector machines) to extract information on physical activity identity from the essays and, second, used that variable to predict the physical activity in adulthood with a separate logistic regression model. The drawback of this approach is that it needs a subset of the data set to have labels for the intermediate outcome (whether physical activity was mentioned in the text or not), which can be time-consuming and labor-intensive for large data sets. Once some labels are available and the prediction model has reasonable performance, the approach can handle large amounts of data to classify the rest of the essays. We have demonstrated that large language models can be directly applied on the data without first generating new intermediate labels.

The major difference between the study by Pongiglione et al [19] and ours, and in general between many epidemiological and data science approaches, is whether the focus is on the causal understanding of associations (etiology) or on prediction. Although the 2 approaches require different study designs and interpretation, the conflation of etiology and prediction is still common in clinical research (eg, causal interpretation of strong predictors) [23]. Our study showed that despite identifying variables strongly associated with the outcome, overall predictive performance might be poor. Therefore, we should be careful when interpreting and drawing causal conclusions from the results of models developed with a predictive aim and avoid mistakenly stating that altering the level of a component of a predictive model would change the risk of the outcome.

Similar evidence also exists regarding the prevention of obesity. In a meta-analysis of 15 prospective studies, Simmonds et al [24] reported that children or adolescents with obesity were about 5 times more likely to be obese in adulthood than those without obesity. In our study, we also found a strong association between BMI in childhood and adulthood; however, the linear regression model performed only slightly better than the naive benchmark, whereas the NLP approach did not outperform the benchmark at all. We were not surprised that NLP performed worse than regression, considering that these approaches had matching performance in predicting physical activity, and obesity was not expected to be directly mentioned in the essays, in contrast to physical activity. In general, the results for this outcome strengthen our previous argument that prediction can be difficult even if well-established associations are present at the population level.

The development of prediction models, regardless of the use of ML or classical methods, is not a trivial task (handling of missing data, variable selection, reporting, etc) [25-28]. This is often reflected in the quality of prediction studies and the fact that only a small proportion of published prediction models are actually used in clinical practice [29]. AutoML does not offer a solution for this, as careful study design is still crucial. However, it makes the use of deep learning techniques (including pretrained models) more feasible for epidemiologists, who can use their resources on study design instead of programming tasks. Faes et al [10] recently reported a study where physicians (non-AI experts) achieved similar performance to expert-tuned algorithms in several medical image classification tasks [10]. We only needed to use programming in the NLP analysis to preprocess the essays and for the evaluation of the results, whereas the rest of the process was completed in a browser environment (model evaluation became available in AutoTrain by Hugging Face soon after we finished our analyses). AutoML solutions are often claimed to democratize ML; however, the financial costs are still not negligible. It is indeed a positive development that technical skills and computational resources no longer pose as strong a barrier as before. We should be vigilant that this increased accessibility is accompanied by an increased focus on good study design and research quality. An aspect that AutoML might have a positive influence on is knowledge translation. With the AutoML approach we used, the deep learning model became available right after training and could be used to make predictions for new samples either in the browser or via an application programming interface. The developer can choose to keep the model private or make it public so that the research community can reuse it as a pretrained model, either directly or after fine-tuning, thus potentially leading to multistep, incremental transfer learning.

A major strength of our work is the use of deep learning methods that are currently state of the art in NLP to exploit an innovative data source—in this case, text written by participants in a cohort study. We compared these models with standard methods in epidemiology and discussed similarities and differences between the classical and data science approaches. A strength of deep learning methods in general is the potential reuse of extant trained models. Although the interest in transfer learning is rapidly increasing in clinical research, it is still an almost unknown concept in the epidemiology community, despite some studies demonstrating major benefits, even for tabular data [30,31]. To increase the impact of prediction studies, especially those using ML and deep learning methods, authors should be encouraged to deposit their models on the web and make them openly available. This is a common practice in the data science community, as most developers depend heavily on pretrained deep learning models due to computational requirements. The Hugging Face Model Hub has >50,000 pretrained models, which fits well with the FAIR (findability, accessibility, interoperability, and reusability) principles on reusing digital assets in an open and inclusive manner [32]. In a clinical research setting, even if data accompany publications, which is still rarely the case, sharing resources is almost exclusively restricted to data sets and analysis code.

The children’s essays used in our NLP models were not designed to be used for specific prediction tasks. Our main aim was to demonstrate the use of deep learning–based large language models and to compare them to the classical statistical methods used in epidemiological research. In showing that NLP methods can extract features from these texts that are associated with certain traits, our study points toward the potential for extracting meaningful additional data from other extant free-text data sources. Each text data source will have its own historical peculiarities and specific characteristics. In our case, the essays were written half a century ago by children. The practical utility of the presented models outside the context of the UK 1958 birth cohort is consequently likely limited without transfer learning via fine-tuning for adaptation to a new context. It should be noted that language models are usually trained on texts from the internet (eg, Wikipedia) and, as such, mostly represent texts written in the past few decades. Where older texts are included—for example, from older, digitized books—sources will represent texts selected for publication at the time. In all cases, texts written by children are likely to be severely underrepresented in training sets.

A previous review of the clinical literature found no evidence for ML having better predictive performance than traditional statistical methods [33]. Considering the trade-off in the loss of easy interpretability, in most studies, the use of ML does not offer any benefits as long as clinical researchers mostly work with tabular data. However, the integration of new data sources in epidemiological studies (text, medical images, and time series) is only possible by applying deep learning and often transfer learning, which also gives us the opportunity to reuse knowledge between studies. With regard to NLP, large language models have almost achieved human-level performance for various specific tasks; therefore, it may become possible for open-ended questions or essays to replace or at least complement long questionnaires (eg, on diet) in large epidemiological studies. Moreover, NLP offers computational methods, for example, for the analysis of interview transcripts in qualitative studies, which might contribute to closing the gap between qualitative and quantitative research. Byrsell et al [34] analyzed transcribed emergency calls to detect out-of-hospital cardiac arrests using deep learning, and Fagherazzi et al [35] recently gave an overview of the potential of vocal biomarkers (containing both linguistic and acoustic features) in clinical research and practice. With the large-scale collection of such and other novel data types, potentially in combination with tabular data, the role of deep learning in epidemiological research is likely to increase as well. However, we can only exploit its potential and develop high-quality prediction models for clinical or public health use in close collaboration between the data science and clinical research communities.

## Abbreviations

AI: artificial intelligence
AUC ROC: area under the receiver operating characteristic curve
AutoML: automated machine learning
BERT: Bidirectional Encoder Representations from Transformers
FAIR: findability, accessibility, interoperability, and reusability
ML: machine learning
NCDS: National Child Development Study
NLP: natural language processing
RMSE: root mean square error
